# Editorial: Caregivers of older individuals: reflections about living, health, work and social conditions

**DOI:** 10.3389/fmed.2023.1269361

**Published:** 2023-09-12

**Authors:** Daniella Pires Nunes, Ariene Angelini dos Santos-Orlandi, Tábatta Renata Pereira Brito

**Affiliations:** ^1^School of Nursing, University of Campinas, Campinas, São Paulo, Brazil; ^2^Department of Nursing, Federal University of São Carlos, São Carlos, São Paulo, Brazil; ^3^School of Nutrition, Federal University of Alfenas, Alfenas, Minas Gerais, Brazil

**Keywords:** caregivers, caregiver burden, dementia, literacy, internet-based intervention, psychosocial interventions, bereavement

The integrated care for older people encompasses a series of measures designed to identify and manage both the physical and mental capabilities of these individuals, while providing support to caregivers responsible for providing care. Care for the older adults, in most countries, is provided by informal caregivers (for example, the spouse, children, other relatives, or friends), and women are the main caregivers (1–5).

The act of caring for someone is a complex task, as it involves several changes and adaptations by both the caregiver and the person being cared for. It is common for caregivers to experience, over time, physical and mental health problems, withdrawal from family and social life, restrictions on leisure activities and, consequently, burden and worsening quality of life (3, 4).

In this context, considering the importance of the caregiver in the comprehensive care of the older adults, the complexity of the act of caring and its repercussions on the life of the caregiver-older dyad, we issued a call for the Research Topic “*Caregivers of older people: reflections about living, health, work, and social conditions”* with the aim of gathering up-to-date evidence and encouraging further research to understand the living, health, and care conditions of caregivers of older people, in different cultural contexts, as well as advances in care and educational practices for this public.

This Research Topic incorporates eight articles to inform research and practices about caregivers of older people, including Brief Research Report, Original Research, and Systematic Review. We also highlight the contributions of research from different geographic scopes such as Asia (China, Korea, and Japan), Europe (Portugal), and Americas (Brazil). We are satisfied with the quality and scope of the contributions received, exceeding our expectations.

Most articles explained about caregivers of older people with dementia. Many caregivers do not have the necessary knowledge to offer dignified care to the older adults, and they end up acting in an experimental and improvised way. Wang et al. identified an association between dementia literacy (knowledge of dementia; attitude toward dementia), assessment of care and psychological wellbeing, and suggested that health professionals incorporate information about dementia literacy in health education for caregivers.

On the other hand, the lack of knowledge can encourage caregivers to seek other forms of learning for care, such as using the internet. Teles et al. study with informal caregivers of older people with dementia investigated the reasons why they used the internet, and the most frequent report was the use to search for generic information about dementia. On the other hand, the lowest frequency of reports was to share experiences with other caregivers online.

For the caregiver to offer quality care, he must be well both physically and emotionally. In the study by Cho et al. investigated the determining conditions for depression in caregivers with dementia and found that the gender of the caregiver has an impact on this morbidity, while economic activities are associated with depression in the general population.

Burden is another manifestation of stress resulting from the process of caring for an older person, covering multidimensional aspects that involve biopsychosocial issues. Marinho et al. found that caregivers with dysfunctional families and satisfaction with care had the highest burden scores. In this sense, strengthening family relationships and social support are important tools for improving the caregiver's quality of life.

Family and social relationships based on reciprocity, cooperation, empathy, and compassion are capable of positively impacting the caregiving experience, also having a beneficial impact on the older person's life. Sato et al. explained that a person with training in Humanitude-care tends to have more empathy, that is, skills to respond emotionally and understand the emotions of people with dementia. The authors revealed that humane caregivers showed more empathetic behaviors, evidenced by facial expressions of stimuli and greater brain activity compared to controls.

Other psychosocial interventions that can positively impact the caring process have been identified by Hoel et al.. Such interventions include encouraging older people with dementia and their caregivers to carry out shared activities and promoting structural changes in the environment in which people with dementia live. In addition, training caregivers on strategies to promote the physical, cognitive, and emotional health of people with dementia can contribute to the enrichment of care relationships.

Still on the importance of training, the World Health Organization developed iSupport for Dementia, an online program that aims to provide skills training and support for family caregivers of people with dementia. Ottaviani et al. evaluated the usability and acceptability of iSupport-Brazil and found results that indicated that the system interface is friendly and the content relevant. Caregivers rated the program as very useful and would recommend it to other caregivers as the usability was considered excellent.

Interventions related to the care process should also consider aspects related to mourning, since the death of an older person does not alleviate care stressors. Alves et al. observed that the transition to grief among caregivers can lead to a decline in mental health, suggesting the need for support during this phase.

As the articles published in this Research Topic demonstrate, reflections on the reality of caregivers involve complex interdisciplinary issues that do not end with the results presented here. We hope that reading this topic will awaken the critical awareness of care providers and researchers so that advances in the area are constant.

## Author contributions

All authors contributed to the conception and design of the project. DN wrote the first draft of the manuscript (Writing—original draft, Writing—review and editing). TB and AS-O edited and added sections of the manuscript (Writing—review and editing). All authors contributed to manuscript revision, read, and approved the submitted version.
